# Giant Bilateral Adrenal Myelolipoma

**DOI:** 10.4274/balkanmedj.2016.1749

**Published:** 2017-09-29

**Authors:** Sushilkumar Satish Gupta, Yizhak Kupfer

**Affiliations:** 1 Department of Medicine, Maimonides Medical Center, New York, USA; 2 Department of Pulmonary and Critical Care Medicine, Maimonides Medical Center, New York, USA

A 36-year-old Hispanic female diagnosed with non-classical congenital adrenal hyperplasia and salt wasting disorder on chronic exogenous steroids presented to our emergency department with excruciating bilateral flank pain, 10/10 in intensity, associated with nausea, vomiting, and generalised weakness. On examination, the patient was in hypovolemic shock with BP of 65/45 mmHg, MAP 52 mmhg, and HR 110 b/min. She was resuscitated with intravenous fluids and intravenous corticosteroids. Physical examination was otherwise unremarkable. Haematology and serum chemistry were non-contributory. The patient had been on high dosages of narcotic analgesics due to persistent abdominal pain, which infrequently required hospitalisations. Current and serial contrast computed tomography scans over the past few years revealed progressive growth of the tumour (Figure 1) with concomitant increase in the level of hormones. The 17 hyroxyprogesterone level was 8000 nmol/lit, androstenedione 864 ng/dL, and total testosterone 65 ng/dL. The surgery and endocrinology teams were consulted, and the patient underwent bilateral adrenalectomy. Surgery seemed to be the best option to lessen the risk of malignancy, prevent retroperitoneal haemorrhage, alleviate the pain symptoms, and decrease opiod dependence. Gross morphology and histopathology results confirmed the diagnosis of giant adrenal myelolipoma (Figure 2, 3). The postoperative period was uneventful, and the patient was discharged from the hospital. Written informed consent was taken from the patient.

Adrenal myelolipomas are rare, benign, and biochemically inactive tumours often diagnosed inadvertently when radiological investigations are performed for other medical conditions. They were first described in 1905 by Gierke as a mass lesion of the adrenal gland comprising a mixture of adipocytes and haematopoietic cells (1). Oberling coined the term ‘formations myelolipomatoses’ in the year 1929 to describe these tumours (2). Females are more often affected than males. Most patients are asymptomatic, but some manifest with abdominal pain due to compression of the adjacent structures or spontaneous rupture. The tumours predominantly consist of adipose tissue and haematopoietic cells, which include myeloid and erythroid elements. They are not an extra medullary site of haematopoiesis, as they contain fat and lack bony spicules and reticular sinusoids. Therefore, they are not associated with any haematological disorders. The differential diagnosis includes teratomas, lipomas, and liposarcomas. The majority of the tumours are unilateral and less than 4 cm; however, tumours greater than 8 cm are referred to as giant myelolipomas (3). Therefore, it is crucial to monitor the size of the tumours with follow-up imaging. It is also often noticed in the setting of bilateral myelolipoma that the tumours on the left side are comparatively larger than on the right side, which can be attributed to the space-limiting constraints of the liver on the right side and unrestricted growth on the left side, as seen in our patient.

Small tumours should be managed by annual radiological screening in an outpatient setting; large tumours of a size greater than 7 cm should be removed, as they carry a potential risk of malignancy and retropeitoneal haemorrhage due to spontaneous rupture (4). In our patient, the probable cause of hypovolemic shock could be severe pain and poor compliance with her medications. Following surgery, the patient did not require any analgesics, hormone levels significantly decreased as compared to before surgery with 17 hyroxyprogesterone levels of 972 nmol/lit and androstenedione of 120 ng/dL, and her quality of life improved. The patient is currently being followed as an outpatient with the endocrinologist, and she is on daily oral replacement therapy with fludrocortisone acetate and hydrocortisone. 

## Figures and Tables

**FIG. 1.**
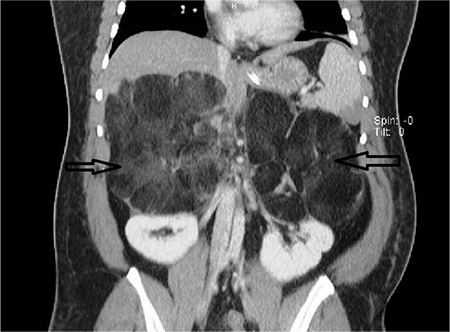
15x13 cm and 16x11.6 cm fatty masses in the region of the right and left adrenal glands, respectively, abutting the kidneys with large feeding vessels.

**FIG. 2.**
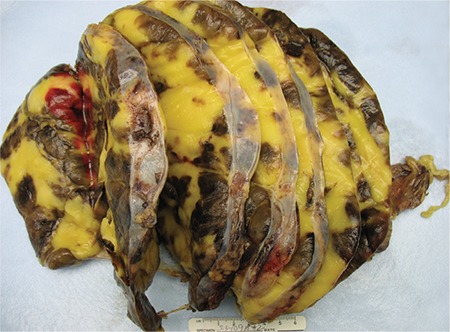
Gross specimen of the adrenal mass.

**FIG. 3.**
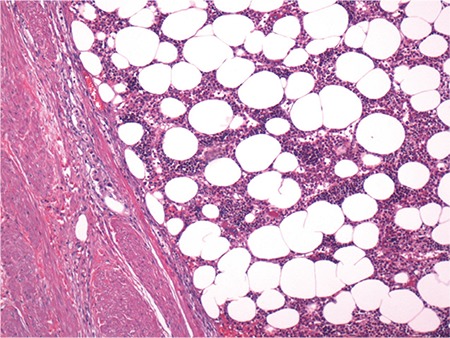
Haematoxylin and eosin stain highlighting large fat globules in the adrenal mass.
